# Surgical Complications Following Appendectomy in Obese Patients: A Single Tertiary Care Center Study

**DOI:** 10.7759/cureus.74033

**Published:** 2024-11-19

**Authors:** Abdulrahman Al Sabr, Abdulrahman Aljohani, Sultan Althoubi, Mohammad Al Marzoqi, Majed Almutairi, Fahad B Al Enizy, Abdullah Al Ghuraibi, Saud Alsadhan, Saud Alrabah, Sami A Boghdadl

**Affiliations:** 1 Medicine and Surgery, King Saud Bin Abdulaziz University for Health Sciences, Riyadh, SAU; 2 General Surgery, King Abdulaziz Medical City, Riyadh, SAU

**Keywords:** appendectomy, appendicitis, bariatric, bmi, complicated appendicitis, laparoscopy, morbidity, obesity

## Abstract

Background

The escalating global prevalence of obesity raises concerns about its implications for health outcomes. While obesity is acknowledged as a major risk factor for various diseases, its impact on appendicitis and appendectomy outcomes remains less explored.

Methods

Data on overweight and obese adults aged 18 to 65 treated for appendicitis in King Abdulaziz Medical City in Riyadh, Saudi Arabia were collected retrospectively. The sample included 1,471 patients who underwent laparoscopic appendectomy between January 2015 and January 2022. The sample did not include patients who were outside the age range, underwent conservative or elective treatment, or were pregnant. Data were collected via the National Guard Health Affairs (NGHA’s) BESTCare 2.0 system. Statistical analyses were performed using the Statistical Analysis System (SAS) (SAS Institute Inc., Cary, NC) software.

Results

The final cohort comprised 564 patients, predominantly male (63.65%) with a mean body mass index (BMI) of 27.41. Comorbidities exhibited varying prevalence among BMI groups, with significant differences observed for diabetes, hyperlipidemia, and hypertension. Notably, 86.35% of the patients did not present with complicated appendicitis or encounter complications, irrespective of BMI. The study found comparable rates of diagnostic CT scan usage across BMI categories. Obese patients displayed a statistically significant trend of longer hospital stays, potentially linked to increased comorbidities and being diagnosed at a later age.

Conclusion

While obesity has been linked to adverse health outcomes, this study found that appendicitis and its surgical management were less influenced by obesity than previously thought. The findings advocate a nuanced approach, acknowledging the impact of obesity on hospitalization trends. This study challenges the assumption that the management of appendicitis in the obese population needs a more tailored intervention.

## Introduction

Obesity has increased significantly in recent years [1]. According to the World Health Organization (WHO), obesity worldwide has almost tripled since 1975 [2]. In 2016, more than 1.9 billion adults were considered overweight and of those, 650 million were obese [2]. This trend can also be seen in Saudi Arabia. In 2020, Saudi Arabia was ranked 14th worldwide on a list of countries with the highest percentage of obesity, with 35.4% of the population being obese [2]. According to a local study, the eastern region of Saudi Arabia had the highest percentage of obesity (29.4%) followed by Riyadh (26.9%), while the city of Al-Baha had the lowest at 14% [3]. This trend in obesity raises concerns regarding comorbidities such as diabetes, hypertension, dyslipidemia, and more [4].

Obesity can be a gateway to other major complications due to its impact on patient hemodynamic status [4,5]. Obese patients experience disturbed platelet functions and estrogen levels, which can increase their risk of deep vein thrombosis events [5,6]. Furthermore, obesity has a major effect on the surgical approach in all three stages: pre-operative, intra-operative, and post-operative [4]. The pulmonary physiology in obese patients is of major consideration for the operative staff during pre-operative surgical workup because the accumulation of fat layers on the neck and chest can obstruct the respiratory airway [5]. Intraoperative challenges include administering anesthesia, placement of intravenous and central lines, and physical strain due to the fatty layers [4]. Postoperative complications include a high risk of poor wound healing and consequent infections [4]. These complications can be seen in multiple operations such as esophagectomy, hepato-biliary injuries, and colorectal surgeries [4].

In contrast to the mentioned surgeries, there is a lack of documentation of appendectomies in the bariatric population. Most often reserved for emergent cases of complicated appendicitis, appendectomies (open and laparoscopic) have been the gold standard practice in medical institutions for many years. Incidences of appendicitis cases globally are estimated to be as high as 229.9 per 100,000 population and in the United States alone there are 300,000 new cases per year [7]. The use of laparoscopy has become increasingly common in appendectomies [8,9]. This study measures the disease and surgical outcomes of bariatric patients in terms of appendectomy due to appendicitis.

## Materials and methods

This is a retrospective cohort study conducted at King Abdulaziz Medical City in Riyadh, Saudi Arabia. The population was 1471 patients who underwent appendectomy from January 2015 to January 2022. Patients younger than 18 or older than 65, those treated conservatively or electively, individuals with a BMI less than 18.5, pregnant patients, and those who underwent appendectomy without surgical findings of appendicitis were excluded from the study sample making the final sample 564 patients. The data were obtained through a chart review using a non-probability consecutive technique. Data on the variables of interest were collected from March through April 2023 using the National Guard Health Affairs (NGHA’s) BESTCare 2.0 hospital information system and accessing patients’ medical record numbers. Patient BMI and age were assessed through these medical records. Surgical procedures and complications were identified based on preoperative assessment notes, including those from the emergency department and surgical ward. Variables related to patients’ healing processes and conditions before discharge were evaluated by following the patients’ postoperative course.

Biostatistics were performed using Statistical Analysis System (SAS) software version 9.4 (SAS Institute Inc., Cary, USA), which is widely used in the field of medical research with robust capabilities for data management and analysis. SAS offers a comprehensive suite of statistical procedures and advanced modeling techniques that are particularly well-suited for handling complex healthcare datasets. For categorical variables, data are presented as frequencies and percentages. Continuous variables are described using the following statistics: n (sample size), n missing, mean, standard deviation, median, quartile range, minimum, and maximum. Chi-squared tests or Fisher’s exact tests were used to determine whether there were statistically significant differences between categorical variables. The Wilcoxon two-sample test was employed to assess associations between continuous data and two-level categorical variables.

## Results

The study sample comprised 564 patients who underwent emergent laparoscopic appendectomy. The sample was 63.65% male and 36.35% female. The mean age was 30 years. Patients were divided into three groups according to BMI. The first group, with a mean BMI of 18.5-24.99 kg/m^2^, included 226 patients. The second group included overweight, with 25-29.99 kg/m^2^, of 182 patients. The third group, obese patients with BMI ≥ 30kg/m^2^, consisted of 156 patients. There was a significant trend with comorbidities, hyperlipidemia, hypertension, diabetes mellitus, and increased weight (Table 1).

**Table 1:** Comorbidities and BMI * indicates that the test used is chi-square (less than 0.05 is significant).

	Normal Weight (BMI 18.5-24.9)	Overweight (BMI 25-29.9)	Obese (BMI ≥ 30)	P-value
Gender to BMI				0.4*
Male	143	122	94	
Female	83	60	62	
Diabetes to BMI				0.04*
Diabetic	24	23	30	
Non-diabetic	202	159	126	
Hyperlipidemia				0.0002*
Hyperlipidemic	17	31	35	
Non-hyperlipidemic	209	151	121	
Hypertension to BMI				0.0003*
Hypertensive	10	14	22	
Non-hypertensive	216	168	134	

Obese patients had the longest length of stay (LOS) in the hospital, and Fisher’s exact P-value was significant (0.0189) (Table 2). There was a diversity of mean age at the time of operation amongst the various groups. Furthermore, on average obese patients who underwent appendectomy were nine years older than normal-weight patients (Table 2).

**Table 2:** Length of stay to BMI and age at time of diagnosis ** indicates that the test used is Fisher’s exact (less than 0.05 is significant). *** indicates that the test used is Kruskal-Wallis (less than 0.05 is significant).

Variable	Normal Weight (BMI 18.5-24.9)	Overweight (BMI 25-29.9)	Obese (BMI ≥ 30)	P-value
Number	226	182	156	
Length of Stay				0.01**
1 day	0	3	0	
2-3 days	160	135	99	
4+ days	66	44	57	
Age to BMI				0.0001***
Mean age at the time of diagnosis	25.92 years	31.52 years	34.21 years

Contrary to expectations, there was no significant correlation between the various BMI groups and appendectomy complications, as 487 patients did not present complicated appendicitis or any intra-procedural or post-procedural complications, chi-square p = 0.7465 (Table 3). The main diagnostic imaging study for all groups was a computed tomography (CT) scan as there was no significant difference in CT usage among the groups (Table 3).

**Table 3:** Disease complication * indicates that the test used is Chi-square (less than 0.05 is significant). ** indicates that the test used is Fisher’s exact (less than 0.05 is significant). (+) means that the patient has a positive variable. (-) means that the patient has a negative variable.

Name	Normal Weight (BMI 18.5 to 24.9)	Overweight (BMI 25-29.9)	Obese (BMI ≥30)	P-value
CT scan				0.4**
CT used	186	143	127	
CT not used	38	39	29	
Complication				0.7*
Complicated Appendicitis	32	22	23	
Uncomplicated Appendicitis	194	160	133	
Perforation				0.5*
Perforated	26	20	23	
Non-perforated	200	162	133	
Intra-abdominal Abscess				0.09*
Intra-abdominal Abscess (+)	14	7	15	
Intra-abdominal Abscess (-)	212	175	141	
Post-Op leakage				0.5**
Post-Op leakage (+)	0	1	0	
Post-Op leakage (-)	226	181	156	
Wound infection				0.7**
Wound infection (+)	1	0	1	
Wound infection (-)	225	182	155	
Post-Op Antibiotics				0.3*
Post-Op Antibiotics (+)	50	33	39	
Post-Op Antibiotics (-)	176	149	117	
Pulmonary Embolism (PE)				1.0**
PE (+)	1	0	0	
PE (-)	225	182	156	
Nosocomial infection				1.0**
Nosocomial infection (+)	2	1	1	
Nosocomial infection (-)	224	181	155	
Re-admission				0.6**
Re-admitted	2	0	1	
Not Re-admitted	224	181	155	

## Discussion

Generally, obesity has negative effects on patients’ outcomes for various diseases and surgeries in comparison to normal BMI. However, our data suggests that patients’ BMI did not significantly influence the management and outcomes of appendicitis and appendectomy.

Obese patients did not have an increased incidence of complicated appendicitis or intraabdominal abscess in comparison to normal weight and overweight patients. The utilization of CT scans for initial diagnosis in the emergency department and post-operative complications showed remarkable similarity across all BMI groups. This uniformity extended to parameters such as nosocomial infections, wound infections, cardiac abnormalities, and post-operative leakage. No significant difference was observed between the BMI groups regarding the need for specialist consultation.

Interestingly, distinctive differences appeared in hospitalization trends. Obese patients demonstrated a statistically significant trend of longer LOS, surpassing four days. This could be attributed to the higher prevalence of comorbidities among the sample of obese patients, including diabetes mellitus, hypertension, and hyperlipidemia. This finding is consistent with the overall notion that obesity causes longer hospital stays in general [10]. However, in studies of hospital LOS among obese patients undergoing appendectomy, the evidence has suggested otherwise [11,12]. The significant findings in our study may be attributed to the larger sample size compared to the smaller cohorts in these previous studies [11,12]. Contrary to our findings, Garey et al. [13] and Knott et al. [14] demonstrated associations of obesity with longer LOS after appendectomy. Those studies also reported longer operative times, increased postoperative analgesics, and worse outcomes in obese appendectomy patients. Clarke et al. [15], in a prospective, randomized double-blind study, found comparable outcomes regarding LOS in obese patients who underwent either laparoscopic or open surgery (mean LOS in days: obese = 4, non-obese = 2, p = 0.140). However, contradictory results have been previously published in several studies [16-19].

While a documentation error could explain the discrepancy, it seems unlikely given the automated nature of the BESTCare system in calculating LOS. The study acknowledges that hospital-specific trends in bed logistics and LOS vary across different hospitals. Given that NGHA is one of the busiest governmental hospitals in Saudi Arabia, its efforts to minimize excess spending and manage bed capacity may lead to a more conservative approach, reducing the likelihood of unnecessary inpatient hospitalizations. Furthermore, variations emerged in the age of appendicitis onset, with obese patients being diagnosed at an older age compared to their normal-weight counterparts.

Limitations

This study population’s characteristics, prevalence of comorbidities, and access to healthcare services may limit the study findings’ generalizability to other populations. It was limited to patients admitted to a single tertiary care center. Moreover, variations in healthcare practices, diagnostic approaches, and surgical techniques across institutions may influence the generalizability of the results. The study does not declare any bias when collecting and analyzing the data. Further research into obesity’s impact on other surgeries may be beneficial in securing an absolute minimum in complications.

## Conclusions

While obesity is known to influence the approach to gastrointestinal surgeries, our study offered a different perspective regarding the impact of patients’ weight on the management of appendicitis. Despite a difference in inpatient LOS and patients’ age, our findings indicate that obesity did not significantly affect the management of appendicitis. This suggests that obese patients with appendicitis can undergo similar management approaches as their non-obese counterparts with confidence. By demonstrating the feasibility of equitable management across BMI categories, our study contributes insights to the field of appendicitis management. These findings have implications for clinical practice, emphasizing the importance of individualized patient care based on specific medical conditions rather than solely on BMI status. Further research may delve deeper into understanding the mechanisms underlying these observations and help improve the treatment and possible stigma when treating obese patients in general.
